# Comparison of heparin to citrate as a catheter locking solution for non-tunneled central venous hemodialysis catheters in patients requiring renal replacement therapy for acute renal failure (VERROU-REA study): study protocol for a randomized controlled trial

**DOI:** 10.1186/1745-6215-15-449

**Published:** 2014-11-19

**Authors:** Rémi Bruyère, Agnès Soudry-Faure, Gilles Capellier, Christine Binquet, Abdelouaid Nadji, Stephane Torner, Gilles Blasco, Maria Yannaraki, Saber Davide Barbar, Jean-Pierre Quenot

**Affiliations:** Medical Intensive Care Unit, University Hospital of Dijon, 14 rue Paul Gaffarel, 21079 Dijon, France; Unité de Recherche Clinique - Réseau d’Aide Méthodologique (URC-ResAM), University Hospital of Dijon, 14 rue Paul Gaffarel, 21079 Dijon, France; Medical Intensive Care Unit, University Hospital of Besançon, 3 Boulevard Fleming, 25000 Besançon, France; Centre d’Investigation Clinique - Epidémiologie Clinique/Essais Clinique (CIC-EC), CIC1432, INSERM, CHU Dijon, Dijon, France; Neuro-traumatological Intensive Care Unit, University Hospital of Dijon, 14 rue Paul Gaffarel, 21079 Dijon, France; Intensive Care Unit of Nephrology, University Hospital of Dijon, 14 rue Paul Gaffarel, 21079 Dijon, France; Surgical Intensive Care Unit, University Hospital of Besançon, 3 Boulevard Fleming, 25000 Besancon, France; Intensive Care Unit of Nephrology, University Hospital of Besançon, 3 Boulevard Fleming, 25000 Besançon, France; LipSTIC LabEx, Fondation de Coopération Scientifique Bourgogne-Franche Comté, Faculty of Medicine, University of Burgundy, 7 boulevard Jeanne d’Arc BP87900, 21079 Dijon, France

**Keywords:** Citrate lock, Heparin lock, Catheter lock, Acute renal failure, Hemodialysis, Critically ill patient

## Abstract

**Background:**

The incidence of acute kidney injury (AKI) is estimated at 10 to 20% in patients admitted to intensive care units (ICU) and often requires renal replacement therapy (RRT). ICU mortality in AKI patients can exceed 50%. Venous catheters are the preferred vascular access method for AKI patients requiring RRT, but carry a risk of catheter thrombosis or infection. Catheter lock solutions are commonly used to prevent such complications. Heparin and citrate locks are both widely used for tunneled, long-term catheters, but few studies have compared citrate versus heparin for patients with short-term, non-tunneled catheters. We aim to compare citrate 4% catheter lock solution versus heparin in terms of event-free survival of the first non-tunneled hemodialysis catheter inserted in ICU patients with AKI requiring RRT. Secondary objectives are the rate of fibrinolysis, incidence of catheter thrombosis and catheter-related infection per 1,000 catheter days, length of stay in ICU and in-hospital and 28-day mortality.

**Methods/Design:**

The VERROU-REA study is a randomized, prospective, multicenter, double-blind, parallel-group, controlled superiority study carried out in the medical, surgical and nephrological ICUs of two large university hospitals in eastern France. A catheter lock solution composed of trisodium citrate at 4% will be compared to unfractionated heparin at a concentration of 5,000 IU/mL. All consecutive adult patients with AKI requiring extracorporeal RRT, and in whom a first non-tunneled catheter is to be inserted by the jugular or femoral approach, will be eligible. Catheters inserted by the subclavian approach, patients with acute liver failure, thrombopenia or contraindication to systemic anticoagulation will be excluded. Patients will be followed up daily in accordance with standard practices for RRT until death or discharge.

**Discussion:**

Data is scarce regarding the use of non-tunneled catheters in the ICU setting in patients with AKI. This study will provide an evidence base for recommendations regarding the use of anticoagulant catheter locks for the prevention of dysfunction in non-tunneled hemodialysis catheters in patients with AKI in critical or intensive care.

**Trial registration:**

Registered with Clinicaltrials.gov (registration number: NCT01962116) on 27 August 2013.

## Background

The incidence of acute kidney injury (AKI) varies according to the populations studied and the definition used, but it is estimated to be around 10 to 20% in patients admitted to the intensive care unit (ICU) [1–3]. The cause of AKI is most commonly acute tubular necrosis occurring in the context of shock (septic and/or cardiogenic), or drug toxicities (such as aminoglycosides or contrast medium). The pathophysiology of AKI is well established, and includes vascular, medullar and tubular injury leading to a reduction in the rate of glomerular filtration, and ultimately, anuria [4]. Renal replacement therapy (RRT) can be required, depending on the AKI etiology and comorbidities, particularly pre-existing renal insufficiency. In one observational study carried out among almost 30,000 ICU patients, the rate of RRT was around 4% [3], but rates as high as 70 to 80% have been reported in patients with shock [5–7]. Mortality in the ICU ranges from 15 to 25%, but can reach 50 to 60% in patients with AKI [3, 8].

Venous catheters are currently the preferred vascular access method for patients with AKI requiring extracorporeal RRT [9, 10]. In critical care, non-tunneled catheters represent standard practice. Despite the vast progress in the management of AKI and high-quality catheter practices [11–13], vascular access remains the weak link in the chain of RRT and contributes to increased morbidity in hemodialysis patients, particularly through catheter dysfunction (stenosis and/or thrombosis) and infection.

Strategies to prevent hemodialysis catheter dysfunction and infection include catheter locks, which are generally either heparin or citrate. The principle of the catheter lock is to instil an anticoagulant solution into the lumen of the branches of the hemodialysis catheter after each RRT session, leaving them in place until the next session.

Heparin locks are considered as the reference for non-tunneled hemodialysis catheters, and are indicated in the setting of RRT. Heparin exerts its anticoagulant effect by binding to antithrombin and antagonizing anti-Xa and anti-IIa activity, as well as all activated coagulation factors. The anticoagulant effect of heparin renders it difficult to handle in the ICU because of the associated bleeding risk [14–16] and the risk of heparin-induced thrombocytopenia [17–19]. Fibrinolysis *in situ* in the catheter has been reported to be necessary in 3 to 9.5 per 1,000 catheter days, reflecting the failure of anticoagulation and the wide variations in heparin concentrations within the catheter that can be observed with this drug [20, 21].

Citrate is an alternative to heparin as a catheter lock solution. Citrate exerts its anticoagulant effect by its ability to chelate calcium, which is fundamental to the activation of the coagulation cascade, but also of platelet activity [22, 23]. Citrate can prevent the formation of a biofilm by calcium and magnesium chelating effects, thus avoiding bacterial colonization [24–28]. Trisodium citrate 4% is considered as the reference lock solution for long-term tunneled catheters in patients undergoing chronic hemodialysis, in order to prevent catheter dysfunction and infection [29, 30]. It is noteworthy that in 2000, the Food and Drug Administration prohibited the use of citrate at concentrations greater than 4% due to the risk of metabolic disorders [31, 32], notably major hypocalcemia resulting in death [33] in cases of systemic leakage. Conversely, bacterial resistance has never been reported with citrate when used as a catheter lock solution.

There are few data from the literature reporting comparisons between citrate and heparin locks for patients with non-tunneled catheters. Data mainly concern patients undergoing chronic RRT with long-term tunneled catheters. These predominantly favored citrate locks for the prevention of infectious and thrombotic risk [28, 34–37].

To the best of our knowledge, only one randomized study, which included 291 patients, has compared the safety and efficacy of heparin versus citrate as a locking solution for non-tunneled catheters in hemodialysis patients [15]. The risk of catheter dysfunction was significantly lower in the citrate group, at 5 versus 8.1 events per 1,000 catheter days for citrate versus heparin respectively (relative risk (RR) 0.38, *P* = 0.015). The authors also noted a lower incidence of catheter infection in the citrate group (1.4 out of 1,000 catheter days) as compared to the heparin group (3.9 out of 1,000 catheter days) (RR 0.36, 95%CI 0.13 to 1.00, *P* = 0.05). Furthermore, the risk of bleeding and death from catheter-related bacteremia was significantly lower in the citrate group. Indeed, it should be noted that this study was prematurely interrupted because of a significant difference in catheter-related bacteremia between groups, in favor of the citrate group. Nevertheless, in this same study, the citrate concentration used was 30% (instead of 4%), and patients in intensive or critical care were excluded.

The primary objective of our study is to demonstrate the superiority of trisodium citrate 4% over heparin as a catheter lock solution, in terms of event-free survival of the first non-tunneled hemodialysis catheter inserted in patients hospitalized in intensive or critical care for AKI requiring extracorporeal RRT. Secondary objectives are to compare the rate of fibrinolysis, incidence of catheter thrombosis and catheter-related infection per 1,000 catheter days, length of stay in an ICU, and in-hospital and 28-day mortality.

## Methods/Design

### Study design

The VERROU-REA study is a randomized, prospective, multicenter, double-blind, parallel-group, controlled superiority study. The study is carried out in the medical ICU, the neuro-trauma ICU and the nephrology ICU of the University Hospital of Dijon, France, and in the medical ICU, the surgical ICU, and the nephrology ICU of the University Hospital of Besancon, France. Two strategies will be compared. Firstly, the treatment under study is a catheter locking solution composed of trisodium citrate at 4% (Citra-Lock, Dirinco AG, Bern, Switzerland). This treatment is not currently approved for use in the context of extracorporeal RRT in critically ill patients with non-tunneled catheters. However, there are international recommendations for its use in chronic dialysis patients with tunneled catheters [29, 30]. Secondly, in the control group, the catheter lock used will be unfractionated heparin at a concentration of 5,000 IU/mL, used within the range of its currently approved indications, namely prevention of coagulation in catheters used for extracorporeal circulation and RRT.

### Ethical considerations

The study protocol was approved by the local independent ethics committee (Comité de Protection des Personnes, Est I) on 21 February 2013 under the number 2013/14 and EudraCT number 2013-000414-37, and by the Agence National de Sécurité des Médicaments et des Produits de Santé (ANSM, French national agency for the safety of medical products and devices, approval number 2013-000414-37). Specific insurance for the study has been contracted by the sponsor from the Hospital Mutual Insurance Company (policy number: 129234). Each patient will provide written informed consent concerning the study design and outcomes. If the information about the study cannot be provided to the patient, his or her relatives will be duly informed about the study.

### Study population

All consecutive patients, hospitalized in any one of the participating units, with AKI requiring extracorporeal RRT will be included in the study. Patients are screened daily by the clinical research monitors and investigators in each center.

#### Inclusion criteria

The inclusion criteria for the study are as follows: patients aged over 18 years, in whom a first non-tunneled catheter is to be inserted by the jugular or femoral approach by medical staff in the participating department.

#### Exclusion criteria

The exclusion criteria are as follows: catheters inserted by the subclavian approach, patients with a contraindication to systemic anticoagulation (active uncontrolled bleeding, acute liver failure (Factor V < 30%), thrombopenia < 30,000/mm^3^ in the absence of planned correction), documented or suspected heparin-induced thrombocytopenia, known allergy to citrate, documented systemic bacterial infection not under treatment at the time of randomization, patients not affiliated with a health insurance system (beneficiary or dependent), pregnant patients or legally protected adults (namely adults in lawful custody or requiring assistance in the conduct of their own affairs or in respect of whom power of attorney is registered).

### Study conduct

Inclusions in the study started on 14 June 2013. All staff likely to be involved in the management of patients included in the VERROU-REA study have attended a training session to undergo training in the monitoring procedures, and posters outlining the main procedures to remember are on permanent display in all participating departments. Time zero is when randomization is performed.

#### Randomization procedure

Randomization will be performed directly through the secure Tenalea™ internet-based software (Formsvision BV, Abcoude, Netherlands) after identification of the investigator by a personal password, which will be communicated prior to initiation of the study. The treatment algorithms have been determined by the study statistician (Clinical Investigation Centre - Clinical Epidemiology/Clinical Trials (CIC-EC), Dijon, France). The allocation is based on a minimization technique taking into account the catheter insertion site (jugular or femoral), type of dialysis (continuous or intermittent) and Simplified Acute Physiology Score (< 55 or ≥ 55) [38]. Patients are assigned to treatment groups (citrate or heparin) using block randomization stratified by center. A comprehensive document describing the randomization procedure is kept in a confidential manner at the CIC-EC of Dijon.

#### Preparation of catheter locks at the hospital pharmacy

Both catheter lock solutions under study are prepared by the pharmacy of Dijon University Hospital and provided to the pharmacy of the participating hospital, with specific labels identifying the study, and in their original packaging, for storage in each participating department in the usual conditions according to the treatment attributed by randomization. The presentation of both solutions (heparin and citrate) is identical (5 mL syringe). Special labels indicating the regulatory information regarding the clinical trials are provided by the pharmacy of the University Hospital of Dijon and stuck to the syringe by the nurse who prepares the catheter lock solution.

Reception, dispensing and return of study drugs will be documented in writing in a dedicated study file kept in the pharmacy. Units of unused study drug will be returned to the pharmacy by the investigator after withdrawal of the first catheter. Since the study is a double-blind design, the investigators are unaware of the type of lock solution being instilled in the catheter for each patient. To preserve the blinding, the lock solution is provided in a box. The preparation to be injected as catheter lock is prepared in a 5 mL syringe by a nurse from the unit, in isolation in a specific room dedicated to the study procedures. The nurse then provides the pre-prepared syringe to the physician in charge of the patient’s management, who injects the lock solution slowly into the catheter lumen. In order to keep the blinding intact, the nurse who prepares the syringe with the catheter lock is not in charge of the patient’s management.

#### Study treatments

Citrate or heparin, according to the study group, will be instilled into each lumen of the catheter to attain a total volume corresponding to the volume of each branch according to the Summary of Product Characteristics. Before each administration of citrate or heparin, the catheter lumens will be flushed with 10 mL of saline as quickly as possible. Then, the lock solution (heparin or citrate) will be injected slowly (over at least 10 seconds duration) into each lumen.

Before initiation of RRT and use of the catheter, a minimum of 5 cc of liquid will be extracted from each lumen (volume greater than the contents of each lumen). Catheter patency will be verified by performing a blood return with a 20 cc syringe. If the 20 cc syringe fills smoothly in under four seconds, this corresponds to a blood flow of 300 mL/min on the hemodialysis machine. All catheters used will have a minimum diameter of 13.5 French. Only one catheter per patient will be considered for the study analysis.

#### Patient management

Daily monitoring of all patients will be performed in accordance with standard practices for patients undergoing RRT. Hemodialysis catheters will be used for the purpose of RRT only (no infusions will be administered through the catheters). For hemodialysis catheter management, two nurses will be present. One will work in aseptic conditions (as for an operating theatre) to manage RRT initiation (withdrawal of lock solution, patency check by blood return and connection of the dialysis lines to the catheter) and completion (remove dialysis lines, flush catheter, insert catheter lock solution and bandage). The second nurse will oversee the control panel and working of the dialysis machine. Hemodynamic monitoring of the patient is performed continuously during RRT, including monitoring of dialysis circuit pressures and transmembrane pressure.

Signs of thrombosis are recorded every time the catheter is connected to the dialysis machine. Signs of infection are recorded several times daily, with measurement of the patient’s temperature every three hours and daily surveillance of the puncture site. Samples will be obtained by swabbing the skin in case of suspected infection, in addition to blood samples. In case of suspected or confirmed infection, the catheter will systematically be removed and the need for antibiotic therapy will be discussed by the medical team. If and when identified, the germs responsible for the infection will be noted. Bleeding complications will also be recorded, defined as a drop in hemoglobin levels requiring transfusion of at least two packs of red blood cells, in cases of major bleeding, and number of packs of red blood cells transfused.

Insofar as possible, the catheter will be removed before patient discharge. Patients will be followed up for the duration of their hospital stay.

#### Data collection

All data will be recorded daily in the dedicated case report form for the entire duration of the study. The following data will be recorded:

1. for each patient: Simplified Acute Physiology Score II, Sequential Organ Failure Assessment score at randomization, demographic data (age, sex, weight, height, comorbidities, clinical and microbiological data to confirm the diagnosis of catheter-related infection (if any)), monitoring (type of RRT, continuous or intermittent RRT, duration of each RRT session and use of systemic anticoagulant) and general complications (bleeding, thrombosis or systemic infection).
2. for each catheter: insertion date, insertion site, catheter removal date, reason for catheter withdrawal, use of ultrasound guidance, experience of the operator (junior, (resident in training), senior with under two years’ experience or senior with more than two years’ experience), need for antibiotic therapy due to suspected or confirmed catheter infection, puncture site bleeding, hematoma, thrombosis, local signs of infection and microbiology (catheter culture, puncture site culture and blood culture).

#### Study definitions

##### Dysfunction

Catheter dysfunction is defined as the inability to achieve and maintain a blood flow of more than 200 mL/min despite changing the patient’s position, inverting the lines and flushing with saline solution. The occurrence of any one or more of these events (such as a change of position, inversion of a line or flush of saline solution) will be considered as catheter dysfunction.

Catheter thrombosis will be treated by the injection of 5,000 to 10,000 IU/mL of urokinase in each lumen of the catheter with a volume equivalent to that of the catheter (noted on each catheter). After 15 minutes, the urokinase will be aspirated with a syringe and dialysis will resume. If urokinase treatment fails to resolve catheter thrombosis, the catheter will be changed [39, 40].

Cather-related infections include the following:

1. Local catheter-related infection, defined as a positive catheter-tip culture of 10^3^ CFU/ml or more, with the presence of pus or oozing at the catheter insertion site, or tunnelitis.
2. General catheter-related infection, defined as a positive catheter-tip culture of 10^3^ CFU/ml or more, with the disappearance of general signs of infection within 48 hours after catheter withdrawal.
3. Catheter-related blood stream infection, defined as the presence of fever (temperature > 38°C) and a positive blood culture taken from the dialysis catheter and a peripheral line in the absence of any other source of infection, with a central-to-peripheral quantitative blood culture ratio of over five, or a differential period of central line culture versus peripheral blood culture positivity for more than two hours, with a central hemoculture showing a positive result more quickly than a peripheral culture.

Systematic samples will be performed at the puncture site in case of any change in skin colour (for example, redness) and/or the presence of pus or oozing suggestive of infection. All samples will be analyzed by the bacteriology laboratory. If infection is confirmed, antibiotic therapy will be initiated. A clinical event committee comprising two physicians (Dr Aho, from the Department of Epidemiology and Hospital Hygiene, University Hospital of Dijon, and Professor Piroth, from the Department of Infectiology, University Hospital of Dijon, France) blinded to the treatment allocation will independently analyze data and adjudicate as to the presence or not of catheter infection. Bleeding complications are defined as a drop in hemoglobin levels requiring the transfusion of at least two units of packed red blood cells.

### Study endpoints

#### Primary endpoint

The primary endpoint is the duration of event-free survival of the first non-tunneled dialysis catheter, defined in days as the time from randomization to catheter withdrawal, whatever the reason (infection, thrombosis, leakage or deteriorated catheter and unintentional or accidental catheter removal).

#### Secondary endpoints

Secondary endpoints are: the rate of fibrinolysis per 1,000 catheter days, the incidence of catheter dysfunction per 1,000 catheter days, the incidence of catheter-related infection per 1,000 catheter days, the number of hemorrhagic events requiring the transfusion of at least two units of packed red blood cells, length of stay in intensive or critical care, length of hospital stay and in-hospital and 28-day mortality rate.

### Sample size calculation

The sample size was calculated using PASS software (version 11, Kayesville, Utah, United States), based on several assumptions. The study by Hermite *et al*. [41] compared the event-free survival of catheters with citrate locks versus saline solution in ICU patients with an indication for extracorporeal RRT. In this study, the median duration of catheter survival with citrate locking solution was 12 days (interquartile range (IQR): 8 to 17], versus six days [3–10] for catheters locked with saline solution (hazard ratio (HR): 2.12, 95% confidence interval (CI): 1.32 to 3.4), *P* = 0.0019). The study by Weijmer *et al*. [15] reported an RR of 0.69 (95%CI: 0.44 to 1.08), *P* = 0.11) for premature withdrawal of non-tunneled, uncuffed catheters with citrate versus heparin. On the basis of these findings, in the prudent hypothesis of a reduction of 25% in the risk of catheter-related events (median of 12 days in the citrate group versus nine days in the heparin group), at an alpha risk of 5% and a beta risk of 20%, a total of 366 patients (183 per group) is required. Accounting for possible crossovers between groups (less than 5% switching from heparin to citrate) due to heparin-induced thrombocytopenia, and allowing for patients lost to follow-up, the final sample size is calculated to be 386 patients (193 patients included per group).

### Statistical analysis

#### Baseline comparability

Quantitative variables will be described as mean ± standard deviation (SD) when normally distributed, or as median (IQR) if non-normally distributed, and qualitative variables as number (percentage). The baseline characteristics of both groups will be compared by univariate analysis (chi square or Fisher’s exact test for qualitative variables, Student’s t-test, Mann-Whitney U test or ANOVA for quantitative variables, as appropriate).

#### Primary objective

The primary analysis will be on an intention-to-treat basis. Event-free catheter survival will be compared between groups using the log rank test. Time zero is defined as the time of randomization, and the time to withdrawal of the first non-tunneled hemodialysis catheter (event-free catheter survival) will be calculated starting from randomization for the analysis of the primary endpoint. Patients will be censored in case of scheduled withdrawal of the catheter, or death. Then, multiple Cox regression analysis will be performed as appropriate, to take into account any potential imbalances in potential confounders between groups. Treatment effects will be expressed as HRs with 95% CI. *P* values will also be provided. A per-protocol analysis will also be performed, using the same techniques. For the per-protocol analysis, patients will be grouped according to the treatment actually received.

An interim analysis is planned once 50% of the expected number of patients has been included (after inclusion of 200 patients). The time between randomization and withdrawal of the catheter will be described in both groups and compared using the log rank test, with an alpha risk fixed at 0.0001 (according to the method proposed by Peto *et al*. [42], to avoid inflating the alpha risk of the main analysis at the end of the study).

#### Secondary objectives

Secondary endpoints will be compared using the chi square test, Fisher’s exact test, Student’s t-test, or the Mann-Whitney U test, as appropriate. Treatment effects will be presented with 95% CI. All analysis will be performed using SAS version 9.2 (SAS Institute Inc., Cary, North Carolina, United States).

### Study organization

#### Data and Safety Monitoring Board

An independent Data and Safety Monitoring Board (DSMB) will monitor the safety and efficacy of the trial and periodically assess whether the trial should continue to the planned termination. Based on the safety data, the DSMB may recommend modifications to the protocol (for example amendments or termination of the study), and, when needed, the DSMB will decide on stopping rules. They will declare any conflicts of interest if such should arise. The DSMB will report to the chairman of the steering committee, who in turn is responsible for implementing a decision to terminate the trial prematurely if deemed necessary.

## Discussion

This study will provide an evidence base for recommendations regarding the use of anticoagulant catheter locks in the prevention of dysfunction in non-tunneled hemodialysis catheters in patients in critical or intensive care with AKI. Current literature is sparse on this topic, with only one randomized study to date [41].

Two types of hemodialysis catheters are used in clinical practice, firstly, temporary catheters (non-tunneled), whose use is limited to a few days, and secondly, long-term catheters (tunneled), which can be left in place for up to several months. In the context of this study, we will only deal with temporary, non-tunneled catheters. Indeed, tunneled catheters can be left in place for longer periods, and are thus more suitable for use in chronic hemodialysis patients. We chose to limit our analysis to non-tunneled catheters, which are inserted for shorter time periods and are mainly used in the setting of AKI. Catheters with these features meet the needs of urgent RRT and are suitable for short-term use because they are quick and relatively easy to put in place [43], and they currently represent standard practice in critical care. There is abundant literature regarding the optimum management of tunneled catheters in patients undergoing long-term hemodialysis, but data is scarce regarding the use of non-tunneled catheters in the ICU setting. For this reason, we focused our investigations on patients with AKI, and not patients requiring long-term RRT.

Although heparin is approved for use as a catheter lock in France, there is insufficient evidence in the literature regarding its safety and efficacy in patients in the critical or intensive care settings. Conversely, there are ample reports concerning the use of trisodium citrate 4% as a catheter lock in tunneled catheters in long-term hemodialysis patients, to prevent catheter dysfunction and infection [28, 34–37]. Thus, it would appear logical that citrate could hold promise for the patient population in our study, notably for those with a contraindication to heparin. Yet, citrate is not approved for use in this indication in France.

In the ICU setting, there are a number of different definitions of dialysis catheter dysfunction, and almost all are applicable in patients on intermittent RRT [41, 44]. In clinical practice, temporary dialysis catheter dysfunction can be defined as the failure to attain and maintain blood flow through the dialysis catheter sufficient for the administration of an adequate RRT dose [45]. Dysfunctions of this type can give rise to infectious complications [46, 47] and may even require that the catheter be changed. In the literature, the incidence of dysfunction depends largely on the type of catheter being used, its size, the anti-thrombotic catheter lock, the patient’s characteristics, the type of therapy and catheter maintenance [48]. In a study by Hryszko *et al*., the frequency of catheter dysfunction was 31.5% [44]. In the French Cathedia study, when only first dialysis catheter placement was assessed, the rate of dysfunction was 10.7% [49].

Infection of the dialysis catheter is a major challenge and can be responsible for 50 to 70% of catheter withdrawals [15, 36]. The incidence of infectious complications in dialysis catheters varies between reports, depending on the catheter type and the population studied. However, it is estimated that the incidence of catheter-related infections (including local infection and bacteremia) is between two and 14 events per 1,000 catheter days, with a median of six [15, 41, 49]. Repeated handling and catheter replacement over a metal guide in case of dysfunction increases the risk of infection [46, 47]. Catheter-related infections are associated with increased length of stay, increased risk of death and higher cost of care [50, 51]. Infections can be prevented by the strict respect of aseptic conditions at the time of catheter insertion.

A limitation of our study is that no analysis of the costs related to the use of citrate as a catheter lock solution (such as cost-efficacy, cost-effectiveness, absolute costs) is planned. Our working hypothesis is that trisodium citrate 4% will be shown to be superior to heparin in terms of risk of catheter dysfunction in non-tunneled hemodialysis catheters. The potential implications of this study are clearly important in the fields of critical and intensive care.

## Trial status

The trial is currently recruiting patients. The recruitment process started on 14 June 2013 and at the time of submission, 191 patients have been recruited. The estimated duration of the inclusion period is 24 months.

## Abbreviations

AKI: Acute kidney injury
CIC-EC: Clinical Epidemiology/Clinical Trials
DSMB: Data and Safety Monitoring Board
HR: Hazard ratio
ICU: Intensive care unit
IQR: Interquartile range
RR: Relative risk
RRT: Renal replacement therapy.
